# Assessing Familiarity, Usage Patterns, and Attitudes of Medical Students Toward ChatGPT and Other Chat-Based AI Apps in Medical Education: Cross-Sectional Questionnaire Study

**DOI:** 10.2196/63065

**Published:** 2025-01-30

**Authors:** Safia Elwaleed Elhassan, Muhammad Raihan Sajid, Amina Mariam Syed, Sidrah Afreen Fathima, Bushra Shehroz Khan, Hala Tamim

**Affiliations:** 1 College of Medicine, Alfaisal University, Takhasussi street, Riyadh, 11533, Saudi Arabia, 966 559441589

**Keywords:** ChatGPT, artificial intelligence, large language model, medical students, ethics, chat-based, AI apps, medical education, social media, attitude, AI

## Abstract

**Background:**

There has been a rise in the popularity of ChatGPT and other chat-based artificial intelligence (AI) apps in medical education. Despite data being available from other parts of the world, there is a significant lack of information on this topic in medical education and research, particularly in Saudi Arabia.

**Objective:**

The primary objective of the study was to examine the familiarity, usage patterns, and attitudes of Alfaisal University medical students toward ChatGPT and other chat-based AI apps in medical education.

**Methods:**

This was a cross-sectional study conducted from October 8, 2023, through November 22, 2023. A questionnaire was distributed through social media channels to medical students at Alfaisal University who were 18 years or older. Current Alfaisal University medical students in years 1 through 6, of both genders, were exclusively targeted by the questionnaire. The study was approved by Alfaisal University Institutional Review Board. A *χ*^2^ test was conducted to assess the relationships between gender, year of study, familiarity, and reasons for usage.

**Results:**

A total of 293 responses were received, of which 95 (32.4%) were from men and 198 (67.6%) were from women. There were 236 (80.5%) responses from preclinical students and 57 (19.5%) from clinical students, respectively. Overall, males (n=93, 97.9%) showed more familiarity with ChatGPT compared to females (n=180, 90.09%; *P*=.03). Additionally, males also used Google Bard and Microsoft Bing ChatGPT more than females (*P*<.001). Clinical-year students used ChatGPT significantly more for general writing purposes compared to preclinical students (*P*=.005). Additionally, 136 (46.4%) students believed that using ChatGPT and other chat-based AI apps for coursework was ethical, 86 (29.4%) were neutral, and 71 (24.2%) considered it unethical (all *P*s>.05).

**Conclusions:**

Familiarity with and usage of ChatGPT and other chat-based AI apps were common among the students of Alfaisal University. The usage patterns of these apps differ between males and females and between preclinical and clinical-year students.

## Introduction

ChatGPT is a sophisticated large language model of artificial intelligence (AI) that was created by OpenAI and released to the public in November 2022 [1]. It generates human-like responses to natural language inputs. The users can hold a conversation with the model where they input a prompt and receive a response [2]. It has many applications including email writing, solving math problems, grammar checking, generating answers to complex questions, and more [3]. Other similar chat-based AI apps include Google Bard, Microsoft Bing ChatGPT, Socrative by Google, Hugging Chat, Snapchat AI, Perplexity AI, and YouChat, among others. All these apps are similar to ChatGPT in terms of generating natural responses to prompts [4].

There has been a rise in new literature pertaining to the use of ChatGPT and other AI tools among medical students. Many published articles show that medical students have a positive attitude toward using ChatGPT in education [5-8]. Many students are eager to use AI tools as they believe it can revolutionize medicine and dentistry [9,10]. Additionally, ChatGPT and other chat-based AIs are continuing to evolve to expand their scope of usage, for example, making virtual histology slides for interactive learning [11-13]. Moreover, ChatGPT can be used in medical education and medical specialties [14]. Its implications in the cardiovascular, cerebrovascular, and radiology fields are being extensively studied, as it can interpret medical imaging and potentially provide a diagnosis [15-17].

Regarding medical research, ChatGPT and similar AI apps can expedite the writing processes by enabling authors to allocate their time and resources more efficiently, by reducing the time spent on the laborious process of searching for relevant literature [18].

A few studies have been conducted to determine students’ willingness to integrate AI tools such as ChatGPT into education. One study demonstrated that both undergraduate and postgraduate students in Hong Kong had a positive attitude toward integrating AI tools into higher education due to its ability to provide immediate solutions, help generate ideas, and handle tedious tasks, allowing students to focus on more important work [6]. Similarly, another study performed on students and faculty at Texas University showed a favorable perception of ChatGPT usage. The responses highlighted the benefits of having access to an AI instructor, which can assist in simplifying concepts by providing examples, offering study advice, and working with students on individual projects [5]. However, the studies were relatively recent and recommend further research, targeting different majors to understand the specialized use of AI in different fields.

Within the Middle East, limited recent studies have assessed medical students’ knowledge and attitudes toward AI. A recent study assessed the awareness, perceptions, and opinions of pharmacy undergraduate students toward AI at King Saud University in Riyadh. The findings indicated a generally positive attitude, with demographic factors such as gender and year of study influencing their perceptions [19]. Another qualitative study investigated the knowledge, benefits, concerns, and limitations associated with the use of ChatGPT among medical college faculty and students in Saudi Arabia; the results highlighted both positive aspects such as enhanced communication and learning, and concerns regarding reliability and privacy [20]. Another study conducted at the University of Jordan involving 623 randomly selected medical students demonstrated a strong positive inclination toward using ChatGPT for learning. The findings recommended integrating ChatGPT into the university curricula, emphasizing benefits for students and the potential for misuse [21].

Due to the rise in popularity of ChatGPT and other chat-based AI in medical education, further research must be conducted to understand students’ familiarity, usage habits, and attitudes toward these technologies. Despite data from other parts of the world and colleges, there is a significant lack of information on this topic in medical education and research, especially in Saudi Arabia. Therefore, this study was designed to study the familiarity, usage, and attitudes of medical students at Alfaisal University toward ChatGPT and other chat-based AI apps for medical education and research. Furthermore, it explores the perceived limitations, advantages, and ethical concerns that arise from their use. This paper addresses the research question “What are the familiarity, usage patterns, and attitudes of Alfaisal University medical students toward ChatGPT and other chat-based AI apps in medical education?” Based on existing literature, we hypothesize that Alfaisal University medical students are familiar with chat-based AI apps and hold positive attitudes toward their use.

## Methods

### Study Design and Enrollment

This study was a closed cross-sectional survey that was conducted among medical students at Alfaisal University. Alfaisal University is a private university in Riyadh, Saudi Arabia, that has around 1500 enrolled medical students.

Only current Alfaisal University medical students in years 1 through 6, of both genders, aged 18 years and above were targeted by the questionnaire; students who did not meet the eligibility criteria were not included in the study. The target sample size was calculated to be in the range of 290‐310 students to achieve a 95% confidence level with a 5% CI, using a sample size calculator.

The online questionnaire was made using Google Forms, a web-based tool used to distribute surveys. The survey was open for responses over 6 weeks, from October 8, 2023, to November 22, 2023. The current survey was modified based on earlier published research [5,6]; the published surveys were chosen in accordance with the IDEE (Identify, Discern, Ethics, Engage) framework, which evaluates how students utilize chat-based AI to achieve specific educational goals, assesses the perceived level of AI integration, examines the effectiveness of AI tools, and explores the ethical considerations involved. The survey was revised to align with our requirements and complement the goals of the study, as previous articles targeted different populations. The answer choices were adapted to reflect the context specific to medical students. The survey was sent to students via email and through Whatsapp groups and other social media outlets including Instagram and Twitter. The survey was designed in accordance with the Checklist for Reporting Results of Internet E-Surveys (CHERRIES) [22,23].

The questionnaire (Multimedia Appendix 1) consisted of 21 questions distributed over 5 pages to assess familiarity, usage, and attitude of medical students toward ChatGPT and other AI apps. The survey consisted of four sections. The first section addressed demographic aspects, including gender (males and females) and academic year (preclinical: years 1‐3; clinical: years 4‐6). The second section had questions regarding the knowledge and use of ChatGPT and other chat-based AI apps, including familiarity, frequency of use, and purposes of usage. Participants were asked to rank their familiarity and frequency through Likert-scale questions. For the purpose of usage, the questions were divided according to uses in medical education and medical research. Participants were asked to select all the relevant choices. In the third section, participants were asked to rate their attitudes toward using ChatGPT or other chat-based AI apps in medical training using Likert-scale questions. They rated their beliefs about the enhancement of medical education through such tools, and their intentions to incorporate them into their future learning practices. The final section investigated ethical considerations of students, including concerns about academic dishonesty.

Descriptive statistics were performed to describe the level of familiarity, reasons for usage, and attitudes of students toward ChatGPT and other chat-based AI apps. A *χ*^2^ test was conducted to assess the relationships between gender and year of studies in terms of familiarity and reasons for usage of ChatGPT and other AI apps. Data analysis was carried out using SPSS (version 29.0; IBM Corp). Statistical significance was set at *P*<.05.

### Ethical Considerations

This study received ethical approval from the Institutional Review Board at Alfaisal University (approval number: IRB-20247). The participants were informed of the purpose of the study; the survey was 4‐5 minutes long and the principal investigator’s email was provided for inquiries. The students provided written informed consent to participate in the research. Participation was voluntary and the students were not given any compensation. To maintain confidentiality, no personally identifiable information such as names or college identity numbers were gathered. The responses were only available to the primary investigators and coinvestigators, and data were anonymized.

## Results

In total, 293 responses fit the inclusion criteria, 95 (32.4 %) of which were from men and 198 (67.6%) from women. There were 236 (80.5%) responses from preclinical and 57 (19.5%) from clinical students, respectively. Participant familiarity with various AI apps is summarized in Table 1. Most students were familiar with ChatGPT and other chat-based AI apps. However, men used ChatGPT, Google Bard, and Microsoft Bing ChatGPT significantly more than women (all *P*s<.05). Additionally, Socrative by Google was used more by students in the preclinical years when compared to students in clinical years (*P*=0.11) (Table 1).

**Table 1.** Familiarity of students toward various artificial intelligence (AI) apps.

AI apps	Total number of responses (N=293), n (%)	Gender			Academic year		
		Men (N=95), n (%)	Women (N=198), n (%)	*P* value	Preclinical (N=236), n (%)	Clinical (N=57), n (%)	*P* value
ChatGPT	273 (93)	93 (97.9)	180 (90.9)	.03	218 (92.4)	55 (96.5)	.27
Google Bard	63 (21.5)	32 (33.7)	31 (15.7)	<.001	50 (21.2)	13 (22.8)	.79
Microsoft Bing ChatGPT	89 (30.4)	41 (43.2)	48 (24.2)	<.001	73 (30.9)	16 (28.1)	.67
Socrative by Google	41 (14)	13 (13.7)	28 (14.1)	.92	39 (16.5)	2 (3.5)	.01
Snapchat AI	181 (61.8)	59 (62.8)	122 (61.6)	.85	145 (61.7)	36 (63.2)	.84
Perplexity AI	7 (2.4)	3 (3.2%)	4 (2)	.55	5 (2.1)	2 (3.5)	.54
YouChat	9 (3.1)	1 (1.1)	8 (4)	.17	9 (3.8)	0 (0.0)	.13
Poe-Telegram-Chatsonic-Replika-Huggingchat	14 (4.8)	7 (7.4)	7 (3.5)	.15	10 (4.2)	4 (7)	.38

Reasons for using various AI apps are summarized in Table 2. Men used ChatGPT for technical questions and solving practice questions significantly more than women (both *P*s<.05). Additionally, clinical students used ChatGPT significantly more for general writing compared to preclinical students (*P*=.005) (Table 2).

**Table 2.** Reasons for using various AI apps.

	Total (N=293), n (%)	Gender			Academic years		
		Men (N=95), n (%)	Women (N=198), n (%)	*P* value	Preclinical (N=236), n (%)	Clinical (N=57), n (%)	*P* value
Usage of Chat-GPT/other chat-based apps for medical education							
Asking technical questions	104 (35.5)	44 (46.3)	60 (30.3)	.007	82 (34.7)	22 (38.6)	.59
Asking general knowledge questions/advice on medical issues	113 (38.6)	39 (41.1)	74 (37.4)	.55	86 (36.4)	27 (47.4)	.13
Solving practice questions	84 (28.7)	34 (35.8)	50 (25.3)	.06	73 (30.9)	11 (19.3)	.08
Generating flashcards	34 (11.6)	13 (13.7)	21 (10.6)	.44	29 (12.3)	5 (8.8)	.46
Asking quick questions when stuck on a problem	115 (39.2)	39 (41.1)	76 (38.4)	.66	95 (40.3)	20 (35.1)	.47
Explaining concepts	101 (34.5)	39 (41.1)	62 (31.3)	.10	78 (33.1)	23 (40.4)	.30
Summarizing text	110 (37.5)	32 (33.7)	78 (39.8)	.31	87 (37)	23 (41.1)	.57
Usage of Chat-GPT/other chat-based apps for medical research							
Helping with assignments, making notes, drafting emails	9 (3.1)	3 (3.2)	6 (3)	.95	8 (3.4)	1 (1.8)	.52
General writing	4 (1.4)	2 (2.1)	2 (1)	.45	1 (0.4)	3 (5.3)	.005
Summarizing texts	97 (33.1)	35 (37.2)	62 (31.3)	.32	79 (33.6)	18 (31.6)	.77
Proofreading	59 (20.1)	23 (24.2)	36 (18.2)	.23	48 (20.3)	11 (19.3)	.86
Grammar checking	82 (28)	29 (30.5)	53 (26.8)	.50	67 (28.4)	15 (26.3)	.75
Paraphrasing	121 (41.3)	41 (43.2)	80 (40.4)	.65	91 (38.6)	30 (52.6)	.053
Writing sections of research	46 (15.7)	16 (17)	30 (15.2)	.68	31 (13.2)	15 (26.3)	.02
Generating citations	39 (13.3)	10 (10.5)	29 (14.6)	.33	35 (14.8)	4 (7)	.12
Searching for relevant articles	63 (21.5)	20 (21.1)	43 (21.7)	.90	45 (19.1)	18 (31.6)	.04
Analyzing literature	39 (13.3)	17 (17.9)	22 (11.1)	.11	33 (14)	6 (10.5)	.49

Attitudes and ethical knowledge toward AI apps are reported in Table 3. Notably, the findings showed that 136 (46.4%) of the participants believed using ChatGPT and other chat-based AI apps for coursework was ethical, 86 (29.4%) were neutral, and 71 (24.2%) considered it unethical (all *P*s>.05) (Table 3).

**Table 3.** Attitude and ethical knowledge toward AI apps.

Aspect	Agree/ethical, n (%)	Neutral, n (%)	Disagree/nonethical, n (%)
ChatGPT/other chat-based AI apps can enhance my medical education	171 (58.4)	96 (32.8)	26 (8.9)
In the future, I plan to incorporate ChatGPT/other chat-based AI apps into my learning procedures	149 (50.9)	96 (32.8)	48 (16.4)
ChatGPT/other chat-based AI apps can help me save time in medical research	188 (64.2)	79 (27)	26 (8.9)
ChatGPT/other chat-based AI apps can provide me with unique perspectives that I may not have thought of myself	193 (65.9)	80 (27.3)	20 (6.8)
ChatGPT/other chat-based AI apps can provide me with personalized and immediate feedback for my assignments	180 (61.4)	81 (27.6)	32 (10.9)
I can become overreliant on ChatGPT/other chat-based AI apps	104 (35.5)	78 (26.6)	111 (37.9)
ChatGPT/other chat-based AI apps will enable academic dishonest behaviors	226 (77.1)	54 (18.4)	13 (4.4)
I understand ChatGPT/other chat-based AI apps can generate output that is factually inaccurate	206 (70.3)	64 (21.8)	23 (7.8)
To what extent do you think using ChatGPT/other chat-based AI apps is ethical for coursework?	136 (46.4)	86 (29.4)	71 (24.2)

## Discussion

### Principal Findings

This study investigated the familiarity, usage patterns, and attitudes toward chat-based AI apps among medical students at Alfaisal University, Riyadh, Saudi Arabia. The findings reveal interesting insights into how this technology is integrated into medical education and research.

When evaluating familiarity, it was found that a significant majority of students (>90%) were familiar with ChatGPT, the most popular application. Additionally, male students exhibited a statistically greater familiarity with, and use of certain apps compared to female students. Furthermore, preclinical students were more familiar with Socrative by Google than other AI apps.

For usage, the primary reasons for using chat-based AI were related to medical education, including asking questions, solving practice problems, generating flash cards, and summarizing texts. Nearly 40% of the students reported using AI to ask quick questions when stuck on a problem and explain concepts. While less prevalent, AI was also used for tasks such as summarizing research texts, proofreading, and paraphrasing.

When questioned about attitudes, most students agreed that chat-based AI could enhance learning, save time, and provide unique perspectives. A vast majority of medical students were willing to incorporate ChatGPT and similar AI apps in their learning strategies and believed that it enabled them to save time. It also provided them with unique perspectives and personalized and immediate feedback on their assignments. Despite the positive outlook, a significant portion of students (37.9%) expressed concerns about overreliance on AI; they also had varying opinions regarding the ethical use of AI for coursework. Despite the positive views on chat-based AI for learning, a significant concern emerged among students. Nearly 77% students feared that these AI apps could contribute to academic dishonesty.

### Implications of Findings

The findings have significant implications for medical education. The high awareness of chat-based AI, particularly among male students suggests that integrating technology into early medical education could enhance learning outcomes. The varied app usage between preclinical and clinical students highlights the importance of tailored educational tools at different training stages. Furthermore, students’ comfort in using AI for daily problem-solving underscores its potential to streamline research workflows and enhance study efficiency, emphasizing the importance of incorporating AI literacy and ethical considerations into curricula.

The findings of this study reinforce the idea that the conventional memory-based medical curriculum, which is primarily memory based, must be followed by advancements in AI. This model has been effective for centuries but demonstrates limitations in the context of the AI age, where technology is evolving to assist with information retrieval, data processing, and clinical decision-making. While memory and foundational knowledge remain important, there is an increasing need for critical thinking, problem-solving, and technological literacy.

Competence in the efficient integration and using knowledge from an expanding range of sources, including the ethical use of AI must be taught to aspiring doctors[16,24]. These findings, unique to this study, reinforce the importance of using AI in medical education [9,16,21,25]. However, students also acknowledged the potential for misuse, highlighting the importance of clear guidelines and fostering a culture of academic integrity alongside the integration of AI in medical education.

### Comparison of Literature

Regarding awareness, our findings are similar to a previously published study from Saudi Arabia that assessed the awareness, perceptions, and opinions toward AI among pharmacy undergraduates. Several students had a positive awareness toward AI and its implications in health care [19].

A cross-sectional study on medical and dental students’ perceptions of AI noted a lack of basic AI education in medical and dental schools. Furthermore, raised concerns about AI-competent doctors may replace doctors those less knowledgeable in using AI. This suggests that educational resources are crucial during earlier stages of medical training to keep up with advancements in AI [9]. Additionally, another study showed that pharmacy students deemed it essential to incorporate AI into college curriculum to effectively educate students on apps in the health care field [19].

A study in Canada reported similar results in terms of attitudes toward AI in research. Conducted on Canadian entry-to–health care students, it found that students who were interested in research generally had a more favorable outlook toward AI [26]. This suggests a potential role of AI in enhancing research efficiency.

However, concerns about the overreliance on AI were similarly found in other studies. For instance, a cross-sectional study conducted among pharmacy students in Saudi Arabia found that 46% of students believed that the use of AI reduced the humanistic aspect of health care, while 7.6% believed that AI devalued the medical profession [12]. Similarly, another study conducted on Canadian health care students expressed those concerns that AI could eventually take over their jobs [26].

There were also varying opinions about the ethical use of AI for coursework. A study at the University of Jordan encouraged educators to integrate ChatGPT into medical curricula and teaching practices, while also addressing student concerns and the potential for misuse [21]. Similarly, a cross-sectional study by Weidner and Fischer [27] in German-speaking European countries highlighted the necessity of incorporating teaching AI ethics into the undergraduate medical curricula. This highlights the need for discussion around responsible AI integration in medical education [9,16,25,28].

A previously published study emphasized the potential for misuse, raising concerns that students might rely on ChatGPT to outsource their assessment tasks [29]. Additionally, in a qualitative study conducted at the Faculty of Medicine, Jazan University in Saudi Arabia, respondents expressed ethical concerns related to threats to academic integrity, plagiarism, privacy, and confidentiality issues [7]. Our findings are similar to these studies, highlighting the importance of clear guidelines and fostering a culture of academic integrity [8,30].

### Limitations of the Study

This study focuses on self-reported data, which may not always reflect actual practices and can cause information bias. There may be a chance of selection bias due to convenient sampling. Additionally, the results may not be generalizable to other countries, as cultural differences could lead to varying attitudes and responses in different contexts.

### Conclusion

Overall, this study provides valuable insights into the growing integration of chat-based AI apps within medical education. As technology evolves, it will be crucial to address ethical concerns and ensure responsible use while maximizing the potential benefits for student learning and research.

## Supplementary material

10.2196/63065 Multimedia Appendix 1 Survey.

## Abbreviations

AI: artificial intelligence
CHERRIES: Checklist for Reporting Results of Internet E-Surveys
IDEE: Identify, Discern, Ethics, Engage
