# Supplementary material for: Assessing Familiarity, Usage Patterns, and Attitudes of Medical Students Toward ChatGPT and Other Chat-Based AI Apps in Medical Education: Cross-Sectional Questionnaire Study
Source: JMIR Med Educ. 2025 Jan 30;11:e63065. doi: 10.2196/63065 (PMC11801772; doi:10.2196/63065)
Supplement: Multimedia Appendix 1 [file mededu-v11-e63065-s001.docx]

**Appendix**

**Survey:**

We are a research team from Alfaisal University, College of Medicine. You are invited to participate in our study on "Knowledge, attitude, and practice of Alfaisal University medical students regarding the use of ChatGPT and other chat-based artificial intelligence applications in medical education”.

Our study aims to assess the knowledge, prevalence, and purposes of use of ChatGPT/ other chat-based AI among AU medical students.

This study was approved by Alfaisal University IRB (IRB-20247). Your participation is voluntary, data is being collected anonymously and no identifying information is attached for this online survey, and you can withdraw at any time.

By submitting the survey, you are voluntarily agreeing to participate. Your participation is valued, the survey will take 4-5 minutes to be filled.

If you have any inquiry or concern, please do not hesitate to contact the principal investigator:

[msajid@alfaisal.edu](mailto:msajid@alfaisal.ed)

**Part 1: Demographic**

Are you 18 and above?

- Yes
- No

1.What is your gender?

- Male
- Female

2. Are you a medical student in Alfaisal University?

- Yes
- No

3. What year of medical school are you currently in?

- 1st
- 2nd
- 3rd
- 4th
- 5th
- Internship

**Part 2: Knowledge and Use**

1. How familiar are you with ChatGPT?

- Extremely familiar
- Very familiar
- Moderately familiar
- Slightly familiar
- Not familiar at all

2. Check all other chat-based AI softwares you are familiar with

- Google Bard
- Microsoft Bing Chat gpt
- Socrative by Google
- Hugging Chat
- Snapchat AI
- Perplexity AI
- YouChat
- None of the above
- Other (please specify)

3. Have you made an account and used ChatGPT/ other chat-based AI apps for any reason (personal or educational)?

- Yes
- No

4. How frequently do you utilize ChatGPT/ other chat-based AI apps?

- Daily
- Weekly
- Monthly
- Rarely
- Never

5. In what ways have you used ChatGPT/ other chat-based AI apps for medical education? (select all that apply)

- Asking technical questions
- Asking general knowledge questions / advice on medical issues
- Solving practice questions
- Generating flash cards
- Asking quick questions when stuck on a problem
- Explaining concepts
- Summarizing text
- Have not used
- Other (Please Specify)

6. In what ways have you used ChatGPT/other chat-based AI for medical research? (select all that apply)

- Summarize texts
- Proof-reading
- Grammar checking
- Paraphrasing
- Writing sections of research
- Generating citations
- Searching for relevant articles
- Analyzing literature
- Have not used
- Other (Please Specify)

7. Rate the statement “I understand ChatGPT/ other chat-based AI apps has limitations in its ability to handle complex tasks.”

- Strongly Agree
- Agree
- Neutral
- Disagree
- Strongly Disagree

8.. Rate the statement “I understand ChatGPT/other chat-based AI apps can generate output that is factually inaccurate.”

- Strongly Agree
- Agree
- Neutral
- D
- Strongly Disagree

5. Rate the statement “ChatGPT/other chat-based AI apps can provide me with unique perspectives that I may not have thought of myself.”

- Strongly Agree
- Agree
- Neutral
- Disagree
- Strongly Disagree

6. Rate the statement “ChatGPT/ other chat-based AI apps can provide me with personalized and immediate feedback for my assignments.”

- Strongly Agree
- Agree
- Neutral
- Disagree
- Strongly Disagree

7. Rate the statement “I can become over-reliant on ChatGPT/ other chat-based AI apps.”

- Strongly Agree
- Agree
- Neutral
- Disagree
- Strongly Disagree

**Part 3.2: Ethics**

1.Rate this statement: "ChatGPT/other chat-based AI apps will enable academic dishonest behaviors."

- Strongly agree
- Somewhat agree
- Neither agree nor disagree
- Somewhat disagree
- Strongly disagree

2. To what extent do you think using ChatGPT/ other chat-based AI apps is ethical for coursework?

- Extremely ethical
- Somewhat ethical
- Neither ethical nor unethical
- Somewhat unethical
- Extremely unethicalisagree
- Strongly Disagree

**Part 3.1: Attitude**

1. Rate the statement "I feel comfortable using ChatGPT/ other chat-based AI apps as part of my medical training"

- Strongly Agree
- Agree
- Neutral
- Disagree
- Strongly Disagree

2. Rate the statement "ChatGPT/other chat-based AI apps can enhance my medical education"

- Strongly Agree
- Agree
- Neutral
- Disagree
- Strongly Disagree

3. Rate the statement “In the future, I plan to incorporate ChatGPT/ other chat-based AI apps into my learning procedures.”

- Strongly Agree
- Agree
- Neutral
- Disagree
- Strongly Disagree

4. Rate the statement “ChatGPT/ other chat-based AI apps can help me save time in medical research.”

- Strongly Agree
- Agree
- Neutral
- Disagree
